# Medical oncologists’ willingness to participate in bundled payment programs

**DOI:** 10.1186/s12913-018-3202-y

**Published:** 2018-05-31

**Authors:** Yonina R. Murciano-Goroff, Anne Marie McCarthy, Mirar N. Bristol, Susan M. Domchek, Peter W. Groeneveld, U. Nkiru Motanya, Katrina Armstrong

**Affiliations:** 1 Department of Medicine, Massachusetts General Hospital, 55 Fruit Street, GRB 740, Boston, MA 02114 USA; 2 Department of Medicine, University of Pennsylvania Perelman School of Medicine, Philadelphia, PA USA; 3 University of Pennsylvania Abramson Cancer Center, Philadelphia, PA USA

**Keywords:** Bundled payment, Physician compensation, Breast cancer, Oncology, Payment reform

## Abstract

**Background:**

Bundled payment programs play an increasingly important role in transforming reimbursement for oncologic care. We assessed determinants of oncologists’ willingness to participate in bundled payment programs for breast cancer. We hypothesized that providers would be more likely to participate in bundled payment programs if offered higher levels of reimbursement for each episode of care.

**Methods:**

Oncologists from Florida, New Jersey, New York, and Pennsylvania were identified in the AMA database or by patients listed in state cancer registries. Providers were randomized to receive one of four versions of a survey describing bundled payment programs offering different levels of compensation for the first year of localized breast cancer treatment ($5000, $10,000, $15,000, or $20,000). Physicians rated their likelihood of participation in a bundled program on a Likert scale. Logistic regression was used to analyze determinants of likelihood of participation in bundling.

**Results:**

Among 460 respondents, only 17% of oncologists were highly likely to participate in a bundled program paying $5000 for the first year of care, rising to 41% for the $15,000 program, but falling to 34% for the $20,000 program. Likelihood of participation was higher among oncologists who were male, older, and believed that cancer patients should not be offered high-cost drugs with minimal survival benefit.

**Conclusion:**

Our results suggest that medical oncologists have limited enthusiasm for bundled payments, and higher payments may not overcome resistance to bundling among a substantial proportion of physicians.

## Background

Rising medical costs have motivated initiatives to redesign physician compensation. Bundled payment programs, in which providers receive fixed fees to care for patients during illness “episodes,” have received increasing national attention [1, 2]. While oncology practices have traditionally operated using fee-for-service payment models [3, 4], in 2013 Medicare launched the Bundled Payments for Care Improvement initiative, with plans to transition 50% of payments from volume-based reimbursement to alternative models by 2018 [5, 6]. Early uptake of bundled payment has predominantly been confined to large hospitals, but the long-term success of Medicare’s initiatives will depend upon individual physicians’ willingness to participate [7]. Physicians’ support is especially critical for the Center for Medicare and Medicaid Services (CMS) Oncology Care Model, which reimburses oncologists through bundled payments for episodes of cancer care, while still covering certain services through traditional fee-for-service payment models alongside pay-for-performance incentives [3]. It is the first of Medicare’s large-scale bundling initiatives to offer payment programs directly to solo-practitioners rather than exclusively to hospitals or group practices [6]. The American Society of Clinical Oncology has also detailed potential reimbursement plans that make use of both bundled payment and pay-for-performance models [3, 4, 8].

Prior studies have raised concerns about physician opposition to bundling [1, 2]. At least 10% of health care costs are generated by patients with cancer [8, 9], yet little is known about medical oncologists’ attitudes towards bundled payments. In this study, we report on the results of a survey asking oncologists about their willingness to participate in bundled programs for breast cancer treatment.

## Methods

We surveyed medical oncologists from Florida, New Jersey, New York, and Pennsylvania.

The physicians included were listed in the American Medical Association Masterfile or were identified by cancer patients who were surveyed as part of a larger study of disparities in genetic testing [10–12]. The states included were chosen for the diversity of their populations as well as the ability to recruit patients directly from the Pennsylvania and Florida state cancer registries of the State Departments of Health. Physicians were contacted by email and/or postal mail. Institutional review board approval was obtained. The provider survey response rate was 29.2% using American Association for Public Opinion Research rate 4 [13]. Respondents and non-respondents did not differ significantly by age (*p* = 0.69) or sex (*p* = 0.10). The University of Pennsylvania and Massachusetts General Hospital Institutional Review Boards approved the study, and considered completion of a questionnaire as implicit informed consent.

Four hundred and sixty medical oncologists confirmed that they see breast cancer patients and responded to questions about bundling. Breast cancer care was felt to be an important focus for a study of bundled payments owing to the prevalence of the disease [14]. A desire to understand the utility of bundling for breast cancer has also led to the development of a pilot bundling program focused on this cancer [9]. Providers were randomized to receive one of four survey versions each describing a bundled program paying a specific amount ($5000, $10,000, $15,000, and $20,000) for medical oncology and infusion costs for the first year of localized breast cancer treatment. For example, for providers randomized to receive the version of the survey describing the $5000 bundled payment program, the survey asked “how likely would you be to participate in bundled payment for localized breast cancer if you received a single payment of $5,000 for the first year of treatment of a patient with localized breast cancer? The payment would include all medical oncologist and infusion costs but not drug, imaging or other costs.” Similarly, providers randomized to receive the $10,000, $15,000, and $20,000 versions of the survey were asked how likely they would be to participate in a bundled payment program offering $10,000, $15,000, or $20,000, respectively, for the same list of services for patients with localized breast cancer. There were no differences in the patient scenarios or excluded treatments described between the different versions of the survey. Payment levels were selected based upon expert opinion and were in keeping with published estimates [15]. Subjects recorded their likelihood of participation in bundled programs on a 5-point Likert scale. In accordance with previous studies [1, 2], the primary analysis dichotomized responses into extremely/very likely versus less likely to participate in bundling.

Items regarding bundled payment programs were embedded in a larger 17-item survey as part of a study of disparities in genetic testing [10–12]. The survey collected information on provider demographics, as well as the characteristics of providers’ patient panels, including the percentage of patients who are black, as well as the percentage who have no health insurance or are insured by Medicaid. Items asking respondents to rate their agreement with a series of statements about the costs of care were modeled after previous studies, including: “patients should have access to all effective treatments for their cancer regardless of cost,” [16] “oncologists have a responsibility to balance the potential benefit of a drug with the potential cost of the drug,” “it is only important to consider the costs of treatment if they are not covered by insurance” [1], and “high cost drugs should not be offered to patients when they have minimal effect on survival.”

Logistic regression was used to examine whether level of compensation, physician characteristics, views about costs of care, and patient-panel demographics predicted physicians’ likelihood of participation in bundling. We additionally carried out ordered logistic regressions, with likelihood of participation analyzed as a 5-level dependent variable.

## Results

Sixty-eight percent of oncologists were male. Mean age was 50. Twenty-two percent of providers were extremely or considerably involved in insurance contracting (Table 1).

**Table 1:** Characteristics of study participants

		Medical oncologists (*n* = 460)
Sex	Female	145 (31.5%)
	Male	315 (68.5%)
Age	30–39 y/o	105 (22.8%)
	40–49 y/o	115 (25.0%)
	50–59 y/o	124 (26.9%)
	60–69 y/o	97 (21.1%)
	70–79 y/o	16 (3.5%)
	80–89 y/o	3 (0.7%)
State	Florida	100 (21.7%)
	New Jersey	81 (17.6%)
	New York	154 (33.5%)
	Pennsylvania	125 (27.2%)
Race or ethnicity	Asian	100 (21.7%)
	Black	13 (2.8%)
	Hispanic	20 (4.4%)
	White	305 (66.3%)
	Other	29 (6.3%)
Percentage of provider’s patient panel who is black	0–5%	145 (31.6%)
	6–100%	314 (68.4%)
Percentage of provider’s patient panel on Medicaid	0–5%	210 (45.8%)
	6–100%	249 (54.3%)
Percentage of provider’s patient panel with no health insurance	0–5%	360 (78.4%)
	6–100%	99 (21.6%)
Degree of provider’s involvement in insurance contracting	Extremely or considerably	101 (22.2%)
	Somewhat, slightly, or not at all	354 (77.8%)

The majority of surveyed providers supported ensuring patient access to effective treatments regardless of cost (75%), but a similar number of respondents felt that oncologists have a responsibility to balance the costs and benefits of drugs (78%) (Table 2).

**Table 2:** Medical oncologists’ willingness to participate in bundling and agreement statements about costs of care

Willingness to participate in bundled payment programs paying	Extremely likely/very likely to participate
$5000 (*n* = 123)	17%
$10,000 (*n* = 129)	25%
$15,000 (*n* = 116)	41%
$20,000 (*n* = 92)	34%
Costs of care:	Strongly/somewhat agree
Patients should have access to all effective treatments for their cancer regardless of cost (*n* = 459).	75%
Oncologists have a responsibility to balance the potential benefit of a drug with the potential cost of the drug (*n* = 460).	78%
It is only important to consider the costs of treatment if they are not covered by insurance (*n* = 460).	23%
High cost drugs should not be offered to patients when they have minimal effect on survival (*n* = 457).	60%

The proportion who were extremely or very likely to participate in bundled programs was lowest among providers who received the survey describing a $5000 program (17%), but was higher for the $15,000 (41%) than for the $20,000 program (34%) (Table 2). In the regression and unadjusted ordered logistic regression model, likelihood of participation increased from the $10,000 to the $15,000 program, but not from the $15,000 to the $20,000 program (Table 3, Table 4). The adjusted ordered logistic regression model yielded similar results (OR 2.3, *p* < 0.001 for $15,000 vs. $5000; OR 1.9, *p* = 0.02 for $20,000 vs. $5000).

**Table 3:** Unadjusted ordered logistic regression on likelihood of participation in a bundled payment program

Category	Odds ratio	*p*-value	95% confidence interval
Bundled payment amount			
$5000	Ref		
$10,000	1.78	0.10	1.14–2.76
$15,000	2.62	< 0.001	1.65–4.17
$20,000	2.22	0.002	1.34–3.62

**Table 4:** Logistic regression and ordered logistic regression modeling likelihood of participating in bundled payment program^a^

	Logistic regression			Ordered logistic regression		
Category	Odds ratio	*p*-value	95% confidence interval	Odds ratio	*p*-value	95% confidence interval
Bundled payment amount						
$5000	Ref			Ref		
$10,000	1.60	0.16	0.83–3.07	1.82	0.01	1.16–2.86
$15,000	3.19	< 0.001	1.69–6.03	2.35	< 0.001	1.45–3.79
$20,000	1.94	0.07	0.96–3.93	1.87	0.021	1.01–3.17
Age	1.03	0.005	1.01–1.05	1.02	0.01	1.00–1.04
Gender						
Male	Ref			Ref		
Female	0.48	0.009	0.28–0.83	0.60	0.009	0.41–0.88
Level of involvement in insurance contracting decisions for practice						
Somewhat/slightly/not at all involved	Ref			Ref		
Extremely/considerably involved	1.55	0.10	0.92–2.62	1.04	0.86	0.67–1.61
Views about the costs of cancer care:						
High cost drugs should not be offered to patients when they have minimal effect on survival.						
Somewhat or strongly disagree/neither agree nor disagree	Ref			Ref		
Somewhat or strongly agree	2.09	0.002	1.30–3.38	1.61	0.009	1.12–2.29
Patients should have access to all effective treatments for their cancer regardless of cost.						
Somewhat or strongly disagree/neither agree nor disagree	Ref			Ref		
Somewhat or strongly agree	1.02	0.93	0.61–1.71	0.96	0.84	0.65–1.42
Oncologists have a responsibility to balance the potential benefit of a drug with the potential cost of the drug.						
Somewhat or strongly disagree/neither agree nor disagree	Ref			Ref		
Somewhat or strongly agree	1.01	0.98	0.59–1.73	1.34	0.17	0.88–2.04
It is only important to consider the costs of treatment if they are not covered by insurance.						
Somewhat or strongly disagree/neither agree nor disagree	Ref			Ref		
Somewhat or strongly agree	1.31	0.32	0.77–2.22	1.27	0.26	0.84–1.95

^a^The regression model was adjusted for physicians’ geographic location by US state as well as for characteristics of physicians’ patient panels, including the percentage of patients who are black, have no health insurance, and are covered by Medicaid. Responses to questions about physicians’ patient panels were collected on a 5-point scale (< 1%, 1–5%, 6–20%, 21–50%, 51–100%), and dichotomized as < 5% of patients versus 6–100%

Likelihood of participation was higher among older oncologists (OR 1.03 for each year, *p* = 0.005) and those believing cancer patients should not be offered high-cost drugs with minimal survival benefit (OR 2.1, *p* = 0.002), but was lower among females (OR 0.48, *p* = 0.009).

## Discussion

Understanding physicians’ attitudes toward bundling is critical to the success of current compensation reform efforts. To our knowledge, this study is the first to focus on oncologists’ willingness to participate in bundled payment programs at different reimbursement levels and offers two major findings.

First, a minority of oncologists were interested in participating in bundled payments for breast cancer care. Previous studies estimated that 6–17% of physicians support or are “very enthusiastic” about bundling [1, 2], similar to the percentage of our respondents who would participate in a $5000 program. Second, the proportion of respondents interested in participating in bundled programs increased as the compensation level increased from $5000 to $15,000, but did not increase further with payment above $15,000. This threshold effect raises the possibility that increases in price may not overcome reluctance to participate among a substantial proportion of oncologists.

Participation in bundled payment requires assuming a level of risk as patients’ clinical courses may be unpredictable leading to uncertainty regarding costs [2, 17, 18]. Even among patients with localized breast cancer, like those described in this survey, a variety of disparate treatments may be required and complications may arise. Our survey focused exclusively on medical oncology and infusion costs, which may differ according to a variety of factors including a patient’s tumor size, hormone receptor and Her2/neu status, tumor gene expression status, whether the patient is pre- or post-menopausal, as well as individual patient variability [19]. Bundled payment programs thus require physicians to accept a degree of uncertainty.

Tolerance of risk varies substantially across individual physicians [20–22]. Furthermore, some providers have relatively little understanding of the costs of care [23, 24] and may not feel responsible for managing costs [1]. Our results accord with studies in which providers affirmed the importance of cost-conscious care but opposed restricting access to effective therapies [1, 16, 25]. Also in keeping with previous investigations showing that older physicians were more likely to support withholding costly therapies with little clinical efficacy [1], older oncologists in our study were more supportive of bundling. Unexpectedly, women were less likely to be interested in bundled payment for breast cancer care, an association that has not previously been described to our knowledge. Further research will be needed to understand these patterns.

This study is limited by small sample size and response rate. While our model controlled for such demographic factors as the percentage of patients in physicians’ panels who are black or are uninsured, there may be other differences in practice characteristics, such as academic affiliation, which correlate with attitudes regarding bundling. Our study is further limited by the fact that our survey did not include items to explicitly assess providers’ level of understanding of payment systems. In practice, physicians’ with different levels of understanding of reimbursement systems are likely to be affected by changes to physician reimbursement schemes, and further study is needed to elucidate how education about payment reform impacts physicians’ willingness to accept bundled payments. Our analysis did control for self-reported involvement in insurance contracting and did not find level of involvement in contracting to be a significant determinant of willingness to participate in bundling. As an increasing number of initiatives are launched to reform physician payment, oncologists’ may be further exposed to bundled payment programs and their attitudes may change [1]. Our study focused on providers’ willingness to participate in bundled payment programs for localized breast cancer, and it is unknown whether these results are applicable to other tumor types.

## Conclusion

In summary, this study of oncologists supports the growing body of evidence that physicians have limited enthusiasm for bundled payments and raises the possibility that higher compensation may not overcome resistance to bundled programs among a substantial proportion of oncologists.
